# Acute Pleurisy - Treatment

**Published:** 1873-12

**Authors:** 


Acute Pleurisy, Treatment—*Service of Prof. N. S. Davis.* Regarding the case as one of pleuritic inflammation, complicated with slight pneumonia and rapidly increasing serous effusion, a moderate-sized blister plaster was applied to the lower part of the right side of the chest, and one teaspoonful of the following prescription given every three hours :

| | |
|---|---|
| ℞.—Hydrochlorate of Ammonia, . . | ʒ iij. |
| Tart. Ant. and Pot., . . . . | grs. ij. |
| Sulph. Morph. . . . . . | grs. iij. |
| Tinct. Digitalis, . . . . . | ℥ i. |
| Syrup Liquorice, . . . . | ℥ iij. |
| Mix. | |

This treatment has been followed by a disappearance of all acute pain, a subsidence of the general febrile symptoms, and a disappearance of the blood from the expectoration.

